# Host-Derived Reactive Oxygen Species in the Gut Epithelium: Defence Mechanism and Target of Bacterial Subversion

**DOI:** 10.3390/antiox14101156

**Published:** 2025-09-24

**Authors:** Pranaya Kansakar, Subhadeep Gupta, Amirul Islam Mallick, Brendan W. Wren, Ezra Aksoy, Abdi Elmi, Ozan Gundogdu

**Affiliations:** 1 Faculty of Infectious and Tropical Diseases, London School of Hygiene and Tropical Medicine, London WC1E 7HT, UK; 2 Department of Biological Sciences, Indian Institute of Science Education and Research Kolkata, Mohanpur, Nadia 741246, West Bengal, India; 3 Center for Biochemical Pharmacology, William Harvey Research Institute, Barts and the London School of Medicine and Dentistry, Queen Mary University of London, London EC1M 6BQ, UK

**Keywords:** reactive oxygen species (ROS), NOX, enteric pathogens, host–pathogen interaction

## Abstract

Host physical, chemical, and immune responses constitute well-established defences against bacterial invasion. Recent studies have highlighted the critical role of cellular mechanisms, particularly the production of reactive oxygen species (ROS) in antibacterial defence. This review focuses on ROS generation by mammalian intestinal epithelial cells (IECs) and investigates whether ROS production is host-driven to eliminate bacteria or manipulated by bacteria to suppress or exploit ROS for enhanced internalisation. We examine the activation mechanisms of the NADPH oxidase (NOX) enzyme complex and the resulting ROS production in IECs, which, unlike professional phagocytes, lack the ability to engulf bacteria. The downstream effects of NOX-mediated ROS signalling are discussed in detail. Additionally, we explore the dynamic interplay between host and pathogen, with particular attention to how bacterial infection may disrupt or hijack host NOX-mediated ROS responses. The review concludes with key experimental considerations and outlines future directions in this evolving field. Overall, we present ROS as a double-edged sword, an essential antimicrobial effector that is also susceptible to bacterial subversion, highlighting its potential as a target in novel antimicrobial strategies.

## 1. Introduction

The large surface area of the gastrointestinal (GI) mucosa provides multiple entry points for pathogenic bacteria into host tissues. In response, the gut has developed various defence mechanisms, including physical barriers, immune responses, and cellular mechanisms to combat pathogen colonisation and invasion. Host-generated ROS play a crucial role in bacterial clearance, as bacteria are susceptible to these oxygen species [1]. Once considered mere by-products of respiration, ROS are now recognised for their role in fighting bacterial infections. They are involved in processes such as lysosomal degradation in phagocytes, cellular signalling, and enhancing the effectiveness of antibacterial treatments [2,3].

Pathogenic enteric bacteria typically access the host intestine upon consumption of, or handling of, contaminated food and water, where infection is generally followed by symptoms such as fever, nausea, vomiting, gastroenteritis and either bloody or watery diarrhoea [4]. The most common bacterial causative agents include *Shigella* spp., *Vibrio* spp., *Salmonella* spp., *Escherichia coli* spp., and *Campylobacter* spp; some of which are classified as intracellular, or at least are considered to have an intracellular phase during infection. Therefore, the host immune system has developed several defence mechanisms to combat invaders. There is a consensus that host cell-mediated ROS production occurs in response to bacterial interaction [2,5]. However, the exact molecular mechanisms behind ROS production are currently being investigated.

This review will outline the physiology of ROS in the context of the NOX enzyme family in IECs. We examine how ROS contributes to the elimination of invading bacteria and how bacteria may manipulate ROS production for their benefit. Lastly, potential experimental approaches to better understand host–bacteria interactions will be discussed, providing new directions for future research in gut immunity.

## 2. Reactive Oxygen Species (ROS)

ROS is an umbrella term for highly reactive intermediaries formed from partially reduced oxygen molecules, such as the organic hydroperoxides (OOH^•^), hydroxyl radicals (OH^•^), and superoxide radicals (^•^O_2_^−^), as well as non-radicals such as hydrogen peroxide (H_2_O_2_) that can be reduced to form radicals [3,6,7]. The chemistry of ROS creation, degradation and interaction with other chemical species is further explored in greater detail elsewhere [8]. There are multiple sources of ROS generation in the epithelial cell [9,10], with key contributors being the mitochondrial respiration chain and the NOX family of enzymes in the context of antimicrobial intervention [11,12,13]. The NOX family comprises seven members, NOX 1-5 and DUOX 1-2, which share a conserved structural organisation [14]. Each has a typical catalytic core with six transmembrane helices housing two haem groups and a cytosolic dehydrogenase domain that interacts with the flavin cofactor and NADPH substrate. Despite this shared structure, they differ in their tissue-specific expression and activation mechanism, which results in functional diversity among the isoforms [15,16]. NOX1, the most abundant isoform in intestinal cells [17] and the primary focus of this review, is activated by Rac1, a member of the Rho family of GTPases [18]. The activity of Rac1 is regulated by a series of upstream molecular switches—guanine nucleotide exchange factors (GEFs), which activate Rac1 by promoting GTP binding, and GTPase-activating proteins (GAPs), which inactivate Rac1 by enhancing GTP hydrolysis [19].

**Figure 1:**
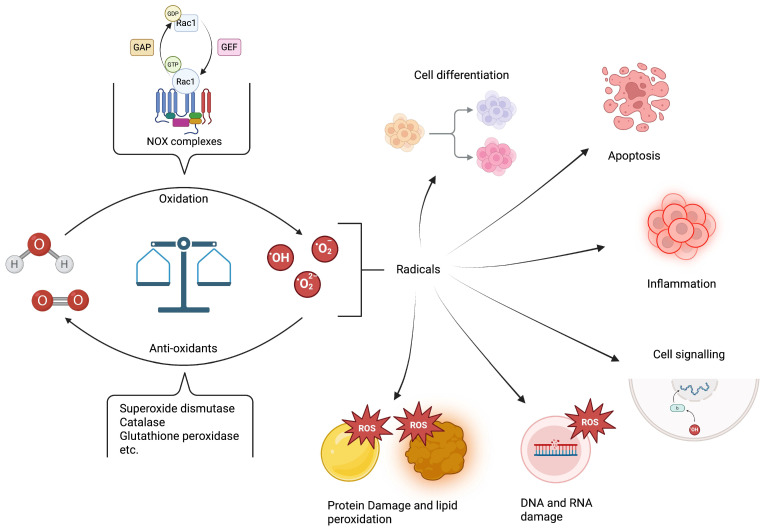
A schematic representation of the balance in ROS mediated by oxidising agents (with examples) and antioxidants, and the downstream effects of ROS. Bacteria here are shown to disrupt the intricate intracellular ROS homeostasis, and are created in BioRender. Kansakar, P. (2025) https://BioRender.com/eehwfh6 (accessed on 18 September 2025).

ROS are short-lived, with typical half-lives of a few microseconds, though this varies depending on the species and the surrounding chemical compounds with which they might interact [20]. Despite this short existence, ROS have many functions, due to their cellular abundance (Figure 1), including cell signalling, immune cell activation and cell fate regulation [10,21,22]. ROS have also been shown to influence the growth and metabolism of gut microbiota, and, conversely, the gut microbiota can modulate ROS concentrations within the vicinity of the gut [23,24]. The effects of ROS depend on their concentration: at lower levels, ROS act as second messengers to activate signalling pathways such as p38/MAPK, JNK1/2, and transcription factors such as NF-κB in both immune and non-immune cell types alike [25,26]. However, at higher concentrations (typically above 100 nM [27]), ROS can damage cells by oxidising proteins and nucleic acids. ROS concentrations often increase several-fold during bacterial infections, suggesting that they help host cells combat intracellular and extracellular bacteria. Numerous antioxidants tightly regulate the intracellular concentrations of ROS in physiological conditions within the picomolar range, to prevent oxidative distress that would otherwise damage cellular components and cellular dysfunction [21].

## 3. ROS in the Context of Host–Pathogen Interactions

### 3.1. The Emerging Role of ROS in Intestinal Epithelial Cell Defence Against Bacterial Invasion

Historically, ROS were mainly linked to the antimicrobial response during phagocytosis in specialised innate immune cells, such as neutrophils, eosinophils, and macrophages, collectively known as professional phagocytes [28]. Recent literature has highlighted novel bacterial clearance mechanisms in non-immune epithelial cells, which lack professional phagocytic abilities. This paradigm shift in understanding suggests that individual cells can independently clear intracellular pathogens, a process known as the autonomous cell response [29]. Key branches of this response include xenophagy, the autophagic degradation of foreign pathogens [30] and bacterial entrapment in septin cages [31], both of which sequester bacteria or render them immobile. ROS, in this context, may complement this autonomous immunity strategy, as they play a role in the clearance of intracellular bacteria in epithelial cells through various mechanisms [32].

**Figure 2:**
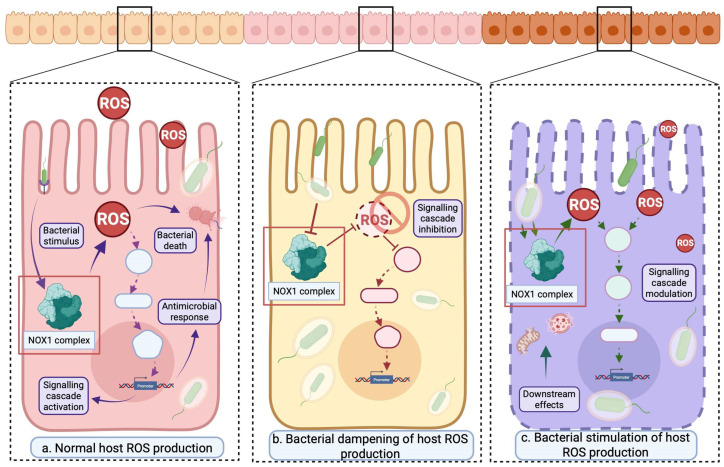
The potential outcomes of bacterial interaction with host cells are (**a**) ROS-mediated bacterial removal, (**b**) bacterial dampening of ROS and subsequent increased prevalence, and (**c**) bacterial stimulation and exploitation of host ROS production. The empty boxes in the diagrams represent generic signalling intermediaries downstream of the NOX1 complex. Solid arrows represent direct events in the different potential outcomes of bacterial interaction with host cells. Dashed arrows represent transient events in signalling pathways. Arrowhead indicates activation. Blunt end indicates inhibition. Created in BioRender. Kansakar, P. (2025) https://BioRender.com/ger6c2g (accessed on 18 September 2025).

There appear to be three predominant scenarios (Figure 2) in which host ROS concentrations can be modified upon bacterial invasion:

- Host-driven ROS elevation in an antimicrobial context;
- Bacterial suppression of host-driven ROS production to evade host defence;
- Bacterial stimulation of host-driven ROS to facilitate host damage and enhance invasiveness.

Thus, the interaction between host IECs and bacteria appears complex and dependent on various factors, such as the species of invading bacteria. Below, we conceptualise these interactions into frameworks based on these scenarios and explore the molecular mechanisms of such interactions between host cells and different bacterial species.

### 3.2. Modulation of Intracellular ROS Concentrations

#### 3.2.1. Bacterial Detection and Host ROS Response

IECs express a broad repertoire of pattern recognition receptors (PRRs) that detect pathogen-associated molecular patterns (PAMPs) and initiate innate immune responses [33,34]. Such receptors include Toll-like receptors (TLRs) that can detect several bacteria-derived molecules, ranging from proteins, lipids, carbohydrates or nucleic acid, to NOD-like receptors (NLRs) that detect peptidoglycan fragments, and RIG-like receptors (RLRs) that detect bacterial RNA. In addition, host IEC receptors such as NOD1 and NOD2 can detect intracellular bacteria through sensing disruption in cytoskeletal components such as Rac1 and actin remodelling [35]. 

A variety of bacterial virulence factors stimulate the host antimicrobial ROS response through interconnected signalling pathways. In *H. pylori* infection, lipopolysaccharide (LPS), acting as a TLR4 ligand, activates the small GTPase Rac1, which directly stimulates NOX1 while also enhancing the transcription of *Nox1* mRNA and its cofactor *NOXO1* [36]. Similarly, in adherent-invasive *E. coli* (AIEC), type 1 pili interact with host cell receptors, including pattern recognition receptors such as TLR4, to promote ROS generation by IECs [37]. Beyond TLR4, TLR1 and TLR2 also contribute by activating MAPK and NF-κB signalling cascades, which regulate *Nox1* transcription [38,39,40]. These pathways converge on NADPH oxidases—including NOX1, NOX2, and DUOX2—linking microbial recognition to oxidative host defence [38]. Notably, the functional outcome of Rac1 activation, at least in neuropathology, depends strongly on its spatial localisation and the nature of the upstream stimulus, which together shape downstream signalling specificity [41]—it remains to be seen if this is the case in bacterial invasion as well. The diversity of bacterial ligands and host PRRs thus likely underlies the variability in epithelial ROS responses across infections, reflecting species-specific strategies by which pathogens invade and manipulate the intestinal epithelium.

Following pathogen recognition by PRRs, IECs rapidly activate intracellular signalling cascades, including pathways leading to the production of ROS [36]. In enteric mucosal cells, *Campylobacter jejuni* infection can trigger Rac1 activation, resulting in increased ROS production [42]. Notably, the term ‘IEC’ encompasses numerous cell types within the luminal barriers of the GI tract, particularly the intestine, each with distinct immunological and physiological functions [43]. While enterocytes are the most abundant at the luminal barrier, serving primarily in nutritional absorption, Paneth cells [44] and goblet cells [45] play unique roles in mucosal defence. Paneth cells reside at the base of the intestinal crypts, where they release antimicrobial compounds. In contrast, goblet cells are interspersed among enterocytes along the crypt–villus axis, secreting mucus into the gut lumen. Given their distinctive immunological roles, how these cell types differ from enterocytes in their ROS responses to bacterial invasion remains to be fully determined. In the following sections, we focus on enterocyte-driven ROS responses as a model for host epithelial defence.

#### 3.2.2. Bacterial Downregulation of the Host Cell ROS Response

Several bacterial species harbour unique mechanisms that suppress host intracellular ROS production during invasion, often by targeting the NADPH oxidase (NOX) system or its upstream regulators. One clear benefit of lowering intracellular ROS is that it creates a more favourable environment for invading bacteria to thrive. A key example of this is the Rac family of GTPases, including Rac1-3, which play an important antimicrobial function, particularly by directly activating NOX enzymes and through other pleiotropic roles in regulating actin cytoskeleton remodelling, cell–cell junction maintenance, and the activation of several kinase cascades [46]. Consequently, bacterial suppression of Rac1 may impair multiple cellular defence mechanisms. Rac1 activation, a precursor to NOX1 activation, is a common target for several enteric pathogens. This occurs either by direct Rac1 inhibition or indirect modulation of its upstream regulators, GEFs (guanine nucleotide exchange factors) and/or GAPs (GTPase-activating proteins). The enteropathogenic *Yersinia* spp. (*Yersinia enterocolitica* and *Yersinia pseudotuberculosis*) directly target Rac1 activation in host IECs. YopE is a secreted toxin by these bacterial species that mimics a GAP and is secreted by these bacterial species to increase virulence. *C. jejuni* was shown by Hong et al. to reduce host ROS levels by downregulating NOX1, and was hypothesised to be inversely correlated with Rac1 activity, though the specific bacterial virulence factor involved remains unidentified [47].

Beyond targeting Rac1, some bacteria interfere with the assembly of the NOX1 complex at the cell membrane. *Vibrio parahaemolyticus* produces the effector protein VopL that assembles actin into non-functional filaments and prevents Rac1 recruitment to the NOX1 complex [48]. At the same time, *Listeria monocytogenes* releases the listeriolysin O toxin that prevents recruitment of NADPH to the phagosome in immune cells, thereby lowering the host cell’s ability to produce ROS—although this effect is specific to phagocytic, rather than epithelial, contexts.

#### 3.2.3. Bacterial Strategies for Stimulating Host ROS Generation

Several bacterial species paradoxically stimulate host ROS production, primarily through activation of NOX enzymes, despite ROS being a significant bacterial clearance mechanism. Rather than impeding infection, this overstimulation can enhance bacterial adherence and invasion of IECs. This admission gives way to the idea that ROS and its production from host IECs can, in fact, be exploited by bacterial species. *H. pylori*, for instance, releases virulence factors such as CagA [49] and VacA [50], which disrupt host signalling pathways, leading to elevated ROS levels [51], resulting in altered cellular processes including proliferation, cytokine release, DNA damage, and cellular apoptosis [52,53]. The CNF1 toxin released from *E. coli* has been shown to disrupt host signalling pathways by specifically binding to, and permanently activating, Rac1. One downstream consequence of these infections is that the ROS concentrations increase inside the host cell [54], resulting in clinical manifestations such as tumorigenesis along the GI tract. Some bacterial infections result in cellular disruption caused by increased host ROS concentrations, leading to cytotoxicity and apoptosis; typical causative agents include *Clostriodes difficile* and its exotoxin TcdB [55], and *Vibrio vulnificus* and its toxin Rtxa1 [56]. Furthermore, some bacterial species are known to adapt to hostile intracellular ROS conditions by altering the microenvironment—*E. coli* LF82 adapts to elevated ROS by modulating mucin production and IL-8 levels, enhancing its capacity to invade and persist within the host [37]. In certain cases, pathogens gain metabolic advantages by co-opting ROS-derived compounds [57]. In *Salmonella* spp., for example, ROS generated, during inflammation, oxidise thiosulfate, a chemical produced by mucosal detoxification of deleterious hydrogen sulphide via caecal colonic bacteria, into tetrathionate [58]. This is selectively utilised by this pathogen as a terminal electron acceptor, to enhance growth in anaerobic or microaerophilic conditions and to accrue a competitive advantage over the microbiota.

Some pathogens (see Table 1) appear to exploit host ROS production via indirect manipulation of signalling pathways. Although IECs possess conserved ROS-generating responses, most notably through NOX1 activation, certain bacterial species exacerbate ROS output during infection. This could arise through direct mechanisms (e.g., secretion of pro-oxidative effectors) or as indirect consequences of host cytoskeletal remodelling. For instance, the modulation of Rac1, a key regulator of actin polymerisation, is a common strategy employed by invasive bacteria to facilitate uptake. Since Rac1 also serves as a critical upstream activator of NOX1, its bacterial manipulation may inadvertently amplify ROS production. Therefore, what may initially appear as a paradox—bacterial enhancement of antimicrobial ROS—can be recast as either an adaptive strategy or an incidental outcome of host manipulation that pathogens have evolved to tolerate or exploit. Thus, ROS elevation may serve as either an adaptive virulence mechanism or a collateral consequence of host subversion—one that pathogens have evolved to withstand or exploit. Supporting this, species that thrive in oxidative environments often encode enhanced antioxidant defences, a topic explored in the following section.

### 3.3. Downstream ROS Effects in the Context of Intracellular Bacterial Clearance

The downstream effects of ROS are concentration-dependent. ROS can act as secondary messengers in low molar concentrations to activate inflammatory and antibacterial signalling cascades. However, ROS can directly attack several bacterial structures and features in higher concentrations, to decrease viability.

#### 3.3.1. ROS as Key Modulators of Cellular Signalling Pathways

There are two known mechanisms by which ROS-mediated signalling can attenuate bacterial pathogenesis and promote clearance: either initiating various immunological and pro-inflammatory signalling cascades or disrupting bacterial signalling required for pathogenicity. Several studies have demonstrated the importance of ROS-mediated signalling in the context of antimicrobial pathway activation and clearance. For example, IECs co-stimulated with TNFα and IL-17 upregulate levels of NOXO1 and ROS, leading to the downstream activation of the p38, MAPK and JNK1/2 pathways and upregulation of the glycoprotein lipocalin-2 [64]. Lipocalin-2 is a bacteriostatic agent interfering with siderophore-mediated iron acquisition by bacteria [65]. Suppression of ROS signalling by DPI in hepatocytes treated with *Listeria monocytogenes* lowered the upregulation of TNF-α and IL-1β, thereby potentiating bacterial virulence [66]. ROS signalling has been shown to activate pre-existing organ-wide defence mechanisms against invading bacteria. Ware et al. demonstrated, in mouse lung epithelial cells, the essential role of DUOX-mediated ROS production in eliciting the antimicrobial properties of Pam2-ODN, a synergistic cocktail of TLR agonists that stimulates mucosal defences within the lungs [67]. It remains to be seen if ROS can activate similar mucosal defences in the intestines. ROS signalling has also been shown to stimulate the production of antimicrobial compounds within the intracellular cytoplasm [68]. Alternatively, Corcionivoschi et al. suggested that ROS can directly regulate bacterial virulence factors, rather than just stimulating host cell defences [42]. The study showed that disruption to the newly identified BY-kinase network in *C. jejuni* by ROS shuts down a network hub instructed by Gne, a UDP-GlcNAc/Glc-4-epimerase required for synthesising the glycan component of cell surface structures. The resulting loss of key surface glycans, such as the capsular polysaccharide and lipo-oligosaccharide reduces bacterial viability and, subsequently, pathogenicity. It remains to be seen if this paradigm of virulence reduction by ROS extends to other enteric pathogenic species that forego IEC invasion.

#### 3.3.2. Direct Mechanisms of Bacterial Clearance Mediated by ROS

Beyond signalling, there are other methods in which ROS and the NOX enzyme family are known to aid in intracellular bacterial clearance, such as DNA and protein oxidation [69,70]. Deoxyribonucleotides, particularly guanine, are susceptible to ROS oxidation [71], which can further potentiate deleterious mutations within the bacterial genome. RNA is more prone to oxidative damage by ROS as it is (i) more abundant in quantity than DNA; (ii) single-stranded, leaving it more exposed to the environment; and (iii) less associated with proteins compared to DNA, and so it is offered less protection from oxidative stresses [72]. Given the numerous forms of RNA, the adverse effects of its oxidation include several transcriptional and translational consequences, such as the failure of rRNA to produce ribosomes and mRNA mistranslation of amino acids into incorrectly folded proteins. ROS can also trigger lipid peroxidation, compromising bacterial cell membrane integrity and leading to protein disulfide bond damage. These attacks may manifest visibly in morphological changes; *C. jejuni*, for example, transitions from a spiral-like to a coccoid form [73], while *E. coli* alters from bacillus to coccoid forms [74] under oxidative stress. In both studies, cell viability decreased with an increased presence of coccoidal cells. In an investigation into membrane integrity disruption, the novel organotin HLSn1 was shown to increase ROS concentrations in *E. coli* and *Bacillus subtilis,* while seemingly disrupting bacterial membrane integrity [75]. The precise mechanisms of how this occurs have yet to be characterised. Nevertheless, the connection between ROS production and membrane integrity reduction suggests a potential link. ROS may also modify the metabolic processes of intracellular bacteria by slowing energy-intensive functions such as cell division and motility, which are essential to normal bacterial function and virulence.

#### 3.3.3. Bacterial Detoxification of ROS

To withstand the hostile oxidative environments encountered within the host intestinal environment and in epithelial cells, bacteria have evolved several sophisticated strategies to detoxify ROS into less reactive compounds [76]. The first involves direct detoxification through bacterially expressed enzymes. Canonical examples include superoxide dismutase (SOD), which converts superoxide radicals into hydrogen peroxide [77], and catalase, which subsequently breaks down hydrogen peroxide into water and oxygen [78]. Additional detoxifying systems are species-specific: *Clostridioides difficile*, for instance, expresses superoxide reductase, which is upregulated in response to host antimicrobial production [79], while *Salmonella Enteritidis* mounts a multifaceted oxidative stress response, including DNA/protein repair and maintenance of redox homeostasis [80]. Together, these mechanisms neutralise ROS, reducing oxidative damage and promoting bacterial survival. A comprehensive review of the oxidative stress responses in bacteria is given in [81]. Another strategy involves the active exploitation of ROS, particularly hydrogen peroxide, as a metabolic resource. Unlike *C. jejuni*, both *Citrobacter rodentium* and *E. coli* utilize colonocyte-derived hydrogen peroxide to enhance their fitness in the competitive intestinal environment. These species engage two distinct respiratory pathways fuelled by H_2_O_2_, a benefit lost in mice lacking epithelial NOX1 activity. In *E. coli*, exogenous H_2_O_2_ supports growth via AppBCX-dependent respiration in a catalase-dependent manner, whereas in *C. rodentium*, NOX1-derived H_2_O_2_ sustains cytochrome c peroxidase (Ccp)-dependent growth [82,83]. Thus, oxidative stress mediated by NOX1 activity not only shapes the oxidative host defence but also provides a metabolic niche that certain bacteria exploit to establish colonisation near the mucosal surface.

## 4. Recent Progress and Conceptual Shifts

The invasion of IECs by bacteria and the resulting host cell ROS response is a dynamic and multifaceted process that remains a key research focus. As outlined in this review, significant progress has been made in understanding these molecular processes and the subsequent downstream effects; however, it is apparent that there are still gaps in our current knowledge of bacterial host invasion and host defence strategies. Moreover, the known information is unevenly distributed among bacterial species. In contrast, several other fields, such as cancer [84], vascular dementia [85], diabetes [86] and stroke [87], have made substantial advancements in ROS biology, such that the role of ROS dysregulation in disease progression is well established, and targeted strategies to modulate intracellular ROS concentrations are actively being explored. Given the rapid advances in ROS biology across other fields, the study of the role of ROS in infectious diseases must close this gap by addressing fundamental unanswered questions, such as the following:

- How can we quantitatively assess bacterial and host cell functional change upon ROS interaction?
- How does the timing and concentration of ROS affect host–pathogen signalling dynamics?
- How does ROS reshape host and bacterial cell metabolism, and how is this reflected phenotypically?
- What histopathological patterns are linked to ROS-modulated bacterial invasion and cytotoxicity?
- Can we therapeutically intervene in ROS-driven signalling during infection, and how do ROS affect outcomes?
- Can therapeutic intervention attenuate the signalling pathway modulations?

## 5. Future Works: Systems-Level Approaches to Host–Pathogen and ROS Interactions

**Figure 3:**
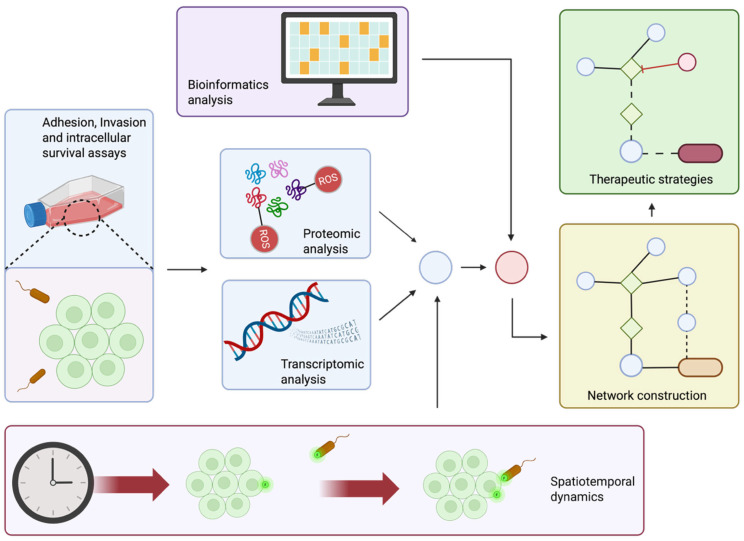
A schematic representation of how different omics approaches can be integrated to create a framework for capturing the network of protein and gene interactions during bacterial invasion. Created in BioRender. Kansakar, P. (2025) https://BioRender.com/8zd78wt (accessed on 18 September 2025).

We outline potential experimental themes that can be conducted in the future to address some of the shortcomings mentioned above (Figure 3).

### 5.1. Multi-Omics Integration for Functional Insights

There have been several recent advancements in multi-omics platforms, particularly in areas such as transcriptomics and proteomics, which enable more informed investigations into the genotypic and phenotypic characteristics of cells. The integration of these technologies allows for a deeper understanding of molecular interconnectivity and enhances our ability to contextualise individual components within the larger biological system. This shift toward systems biology creates a transformative framework that moves beyond isolated molecular analysis to construct dynamic, network-level models. Systems biology reconceptualises experimental data, linking genes and proteins within interconnected pathways and embedding them within the broader biological context, to reveal emergent behaviours and system-wide responses [88].

#### 5.1.1. Transcriptomics for Host–Pathogen Profiling

The establishment of innovative frameworks for genomic analysis, such as gene ontology [89], co-expression network analysis [90], and pathway enrichment analysis [91], allows the dissection of the functional properties of gene products and the synergies of these individual components as they form into gene networks that have profound impacts on cell phenotype. Cutting-edge transcriptomic techniques such as dual-RNA sequencing can resolve gene expression changes by simultaneously sequencing the host and bacterial transcriptome to investigate gene regulation across both systems during the invasion cycle. Though dual-RNA seq is a relatively new technique, it has led to interesting research avenues to elucidate bacterial genes involved in intracellular invasion [92]. Regarding ROS interactions between host cells and enteric pathogens, a recent study utilised dual-RNA seq to identify novel stress responses in *H. pylori* upon invasion of GES-1 gastric epithelial cell lines [93]. Additionally, it was found that the virulence factor CagA disrupts the electron transport chain, and subsequently increases intracellular ROS concentrations [93]. Insights such as these directly contribute to elucidating the precise molecular mechanisms by which bacteria modulate ROS and the host cell responses to bacterial invasion.

#### 5.1.2. Proteomics and Post-Translational Modulation by ROS

Proteomic analysis can be run in tandem to gain even deeper insights into host-–athogen interactions and ROS effects on cellular physiology. Proteins play a diverse role in the intracellular environment, from signalling [94] to bacterial killing [95], independent of transcriptional modifications. Approaches like mass-spectrometry can quantify changes to intracellular levels of antimicrobial peptides and signalling molecules in response to bacterial invasion, and determine whether the effects are influenced by ROS produced in response to bacterial invasion. ROS can also directly modify post-translational modifications [96], altering their function; for example, through the oxidation of amino acids, ROS can change protein conformation and alter binding sites, changing antimicrobial peptide function [97]. Transcriptomic analytical approaches overlook these post-translational modifications, and, therefore, deploying proteomics alongside transcriptomics is critical in developing a more refined picture of host–pathogen interactions.

### 5.2. Imaging-Guided Spatial and Temporal Resolution

While omics-based approaches provide valuable global snapshots of differential gene and protein expression following host cell invasion, they offer only a static and partial view of the host–pathogen interface. Imaging technologies will transform static molecular profiles into dynamic, spatiotemporally resolved models of infection [98]. One problem imaging may overcome is resolving population heterogeneity within intracellular bacterial populations. Bacterial gene expression is tied to distinct stages of the infection cycle [99], suggesting individual bacterial cells may express unique transcriptomes, based on their location in the cell. Bulk sequencing of these bacterial populations may lead to significant variation in datasets, due to their location. Advanced microscopy techniques can uncover phenotypic heterogeneity within bacterial populations [100] and may reveal differences in subpopulation behaviours based on intracellular localisation. In addition to these techniques, commonly used imaging approaches, such as light and electron-based microscopy and their derivatives, are reviewed in [101]. Another imaging concept that can be considered is the spatial and temporal tracking of individual RNA molecules, as reviewed in [102]. This allows for the precise localisation of RNA molecules and their interactions with different RNA and protein molecules over time. By contextualising the ‘when’ and ‘where’ of interactions within cells and tissues, imaging will add a crucial dimensionality that transcriptomics and proteomics alone cannot achieve.

## 6. Concluding Remarks

ROS generated by NADPH oxidases NOX in IECs are pivotal components of the host’s frontline defence against bacterial pathogens. Unlike professional phagocytes, IECs depend on NOX-mediated ROS for antimicrobial signalling without engaging in phagocytosis. Increasing evidence indicates that bacterial pathogens can disrupt or exploit these ROS pathways, to enhance their internalisation and survival. Despite recent advances, significant gaps remain in our understanding of the underlying molecular mechanisms and pathogen-specific strategies involved. Future research employing emerging approaches such as dual-RNA sequencing and proteomics will be critical to disentangling this complex host–pathogen interaction. A deeper understanding of how ROS act, both as antimicrobial effectors and as targets of bacterial subversion, will be essential for the development of novel strategies to strengthen mucosal immunity and combat infection-driven disease.

## Figures and Tables

**Table 1:** A summary of selected enteric/gastric bacterial species that stimulate or dampen host ROS production during invasion.

Bacterial Species	Changes in ROS	Host or Bacteria Driven	Proposed Mechanism of Action on ROS	Cell Lines	References
*Helicobacter pylori*	Increase	Bacteria	Bacterial LPS from whole bacteria activates transcription of NOX1 and NOXO1 genes, and activates Rac1 to increase intracellular ROS	Guinea pig mucosal cell	[59]
*Escherichia coli LF82*	Increase	Host	ROS production is induced by NOX1, and increases NOX1 and NOXO1 gene expression, mostly likely as an antimicrobial response	T84	[37]
*Escherichia coli*	Increase	Bacteria	The toxin cytotoxic necrotisingfactor-1 acts (hypothesised) as a GEF to permanently activate Rac1 and subsequent ROS production	IEC-6 cells (normal rat small intestine)	[54]
*Clostridioides difficile*	Increase	Bacteria	Toxin TcdB induces ROS production via transient activation of Rac1 and subsequently NOX1, leading to IEC necrosis	Young adult mouse colonic epithelial cells	[55,60]
*Vibrio vulnificus*	Increase	Bacteria	The release of toxin RtxA1 acts via NOX1 to overproduce ROS, and also modulates Rac2 activity	Caco-2	[56]
*Campylobacter jejuni*	Increase	Host	Binding via CadF protein leads to NOX1 activation as a defence mechanism	HCT-8	[42]
*Campylobacter jejuni*	Decrease	Bacteria	Unknown bacterial component downregulates NOX1-mediated ROS production. This is hypothesised to correlate with Rac1 activity	Caco-2 and T84	[47]
*Yersinia pseudotuberculosis*	Decrease	Bacteria	The cytotoxin YopE acts as a GAP protein to decrease Rac1 activity	HeLa	[61]
*Listeria monocytogenes*	Decrease	Bacteria	Pore-forming cytolysin listeriolysin O prevents *NOX2* phagosome localisation in phagocytes	RAW 264.7 macrophage	[62,63]
*Vibrio parahaemolyticus*	Decrease	Bacteria	T3SS effector protein VopL paralyses actin cytoskeleton and stops NOX1 complex	Caco-2	[48]
